# TRPV4-Mediated Calcium Influx into Human Bronchial Epithelia upon Exposure to Diesel Exhaust Particles

**DOI:** 10.1289/ehp.1002807

**Published:** 2011-01-18

**Authors:** Jinju Li, Patrick Kanju, Michael Patterson, Wei-Leong Chew, Seung-Hyun Cho, Ian Gilmour, Tim Oliver, Ryohei Yasuda, Andrew Ghio, Sidney A. Simon, Wolfgang Liedtke

**Affiliations:** 1 Department of Medicine and; 2 Department of Neurobiology, Duke University, Durham, North Carolina, USA; 3 U.S. Environmental Protection Agency, Research Triangle Park, North Carolina, USA; 4 Department of Cell Biology, Duke University, Durham, North Carolina, USA; 5 Howard Hughes Medical Institute, Chevy Chase, Maryland, USA; 6 U.S. Environmental Protection Agency, Chapel Hill, North Carolina, USA

**Keywords:** air pollution, COPD, DEP, human bronchial epithelia, MMP-1, PAR-2, PI3-kinase, PLCβ3, TRPV4, TRPV4_P19S_

## Abstract

**Background:**

Human respiratory epithelia function in airway mucociliary clearance and barrier function and have recently been implicated in sensory functions.

**Objective:**

We investigated a link between chronic obstructive pulmonary disease (COPD) pathogenesis and molecular mechanisms underlying Ca^2+^ influx into human airway epithelia elicited by diesel exhaust particles (DEP).

**Methods and Results:**

Using primary cultures of human respiratory epithelial (HRE) cells, we determined that these cells possess proteolytic signaling machinery, whereby proteinase-activated receptor-2 (PAR-2) activates Ca^2+^-permeable TRPV4, which leads to activation of human respiratory disease–enhancing matrix metalloproteinase-1 (MMP-1), a signaling cascade initiated by diesel exhaust particles (DEP), a globally relevant air pollutant. Moreover, we observed ciliary expression of PAR-2, TRPV4, and phospholipase-Cβ3 in human airway epithelia and their DEP-enhanced protein–protein complex formation. We also found that the chronic obstructive pulmonary disease (COPD)–predisposing TRPV4_P19S_ variant enhances Ca^2+^ influx and *MMP 1* activation, providing mechanistic linkage between man-made air pollution and human airway disease.

**Conclusion:**

DEP evoked protracted Ca^2+^ influx via TRPV4, enhanced by the COPD-predisposing human genetic polymorphism TRPV4_P19S_. This mechanism reprograms maladaptive inflammatory and extracellular-matrix–remodeling responses in human airways. The novel concept of air pollution–responsive ciliary signal transduction from PAR-2 to TRPV4 in human respiratory epithelia will accelerate rationally targeted therapies, possibly via the inhalatory route.

Human airway epithelia function in barrier formation, defense against pathogens, and mucociliary clearance (Hogg 2004). They represent the first barrier against airborne environmental pollutants, and they coordinate recruitment of pivotal inflammatory cells in several pathologies, including chronic obstructive pulmonary disease (COPD) (Li et al. 2009; Mercer et al. 2006; Yang et al. 2005). The inhalation of diesel exhaust particles (DEP), produced by vehicular traffic contributing to urban smog, leads to serious respiratory diseases (e.g., COPD, emphysema, bronchial cancer, chronic asthma) (Torres-Duque et al. 2008). The particles’ carbonaceous cores are coated with thousands of organics and heavy metals. Because large numbers of hazardous chemicals are present on DEP, its pathological effects on human airways are pleiotropic. We and others have found that DEP evokes the secretion of matrix metalloproteinase-1 (MMP-1) from human bronchial epithelia (Amara et al. 2007; Li et al. 2009). Matrix metalloproteinase-1 (MMP-1) plays a role in tissue remodeling during development, inflammation, migration of inflammatory and malignant cells, and COPD and emphysema pathogenesis (Segura-Valdez et al. 2000). It also has neurotropic effects, possibly enhancing sensitization of airway-innervating sensory neurons, contributing to airway hypersensitization and chronic cough (Conant et al. 2004). We recently identified a novel pathway that results in DEP-induced *MMP-1* activation and entails activation of RAS-RAF-MEK-extracellular signal–regulated kinase (ERK) signaling, dependent on β-arrestins (Li et al. 2009). From a global health perspective, one important finding was that the human *MMP-1* polymorphism at position −1607(1G/2G) of the *MMP-1* promoter yielded, after DEP exposure, either a diminutive (1G) or large (2G) response. The 2G polymorphism is found in 75% of humans.

Against this background, we sought to identify critical elements upstream of RAS in human airways in response to DEP. The pathogenic component of DEP that activates *MMP-1* is primarily retained in its organic extract (OE), such that DEP carbonaceous core particles shuttle water-insoluble OE to the ciliary plasma membrane. The DEP/OE initially activates proteinase-activated receptor 2 (PAR-2), which, via G_i/o_ G-protein, phospholipase-Cβ3 (PLCβ3), and phosphatidylinositol 3 kinase (PI3-K), activates Ca^2+^-permeable TRPV4 (transient receptor potential vanilloid, family member 4) ion channels (Liedtke et al. 2000; Lorenzo et al. 2008; Sidhaye et al. 2008; Strotmann et al. 2000). A uniquely protracted Ca^2+^ influx through TRPV4 follows, which is critical for mitogen-activated protein kinase (MAPK)–mediated *MMP-1* activation. Localization studies show that PAR-2, PLCβ3, and TRPV4 colocalize to cilia of human differentiated airway epithelia. DEP exposure greatly enhances protein–protein complex formation between these signaling molecules and calmodulin. Importantly, we observed that TRPV4_P19S_, a human genetic polymorphism previously identified as a COPD susceptibility locus (Zhu et al. 2009), increases *MMP-1* activation via increased Ca^2+^ influx, providing a mechanistic link between human airway epithelia signaling, airway disease, and air pollution.

## Materials and Methods

### DEP

Particles were generated at the U.S. Environmental Protection Agency (EPA; Research Triangle Park, NC) from a Deutz four-cylinder diesel engine, running at three defined engine loads before collection, as described previously (Li et al. 2009). For experiments, we used DEP at 100 μg/mL. DEP organic extract (OE) was prepared by washing organic chemicals off of DEP using methylene chloride, followed by solvent exchange with dimethyl sulfoxide (DMSO). In experiments, we used 20 μg/mL OE, which is equivalent to the organic compounds contained in 100 μg DEP. We used Degussa Printex 90 carbon nanospheres (P90; provided by W. Moeller, GSF, Munich, Germany) as controls.

### Chemicals

We used the following compounds: pertussis toxin (G_i/o_ inhibitor; Sigma Chemical Co., St. Louis, MO), U73122 (PLC inhibitor; Tocris, Ellisville, MO), LY294002 and PI828 (PI3-K inhibitors; Tocris), 4α-phorbol 12,13 didecanoate (4α-PDD; TRPV4 activator; Tocris), GSK205 [TRPV4 blocker (Phan et al. 2009)], ruthenium red [TRP(V) blocker; Tocris], gadolinium(III) chloride [GdCl_3_ (Sigma); inhibitor of store-operated calcium entry (SOCE) at 5 μM (Bird et al. 2008)], thapsigargin (Ca^2+^-store depletion; Tocris), GM1489 and Z-PDLDA-NHOH (pan-MMP inhibitors; Endogen, Rockford, IL), and W-7 (calmodulin blocker at 10 μM; Sigma).

### Cell culture

BEAS-2B cells were obtained from ATCC (Rockville, MD), maintained as previously described (Li et al. 2009), and used for stimulation with DEP or OE and for all DNA and small interfering RNA (siRNA) transfection experiments. Primary human bronchial epithelial (HBE) cells were tracheobronchial cells derived from healthy, nonsmoking adult volunteers. We obtained institutional review board approval for this study from the participating institutions, and volunteer donors provided informed consent for use of the cells in research. Additional details on cell culture are available in Supplemental Material (doi:10.1289/ehp.1002807).

*MMP-1* reporter gene assays were conducted as described previously (Li et al. 2009). A set of *MMP-1* promoters of different lengths was available for both human polymorphisms, −1607G and −1607GG.

siRNA was transfected into BEAS-2B cells following previously published methods (Li et al. 2009). siRNA was directed against PAR-2, PAR-1, β-arrestins 1 and 2, and TRPV4. Scrambled controls were used as provided by the manufacturer (Dharmacon, Lafayette, CO). siRNA efficiency was confirmed by quantitative reverse-transcriptase polymerase chain reaction (PCR) and Western blotting.

TRPV3 and TRPV4 dominant-negative (DN) isoforms were generated by isolating a truncated form of each channel, from 10 amino- acids N-terminal to the fifth transmembrane domain to 10 amino-acids C-terminal to the sixth transmembrane domain. In addition, two point mutations were generated as M680K and D682K for TRPV4 and as L619K and D621K for TRPV3 in order to render the channel fragments Ca^2+^ impermeable. Both of these constructs were C-terminally fused to monomeric red fluorescent protein (RFP).

Dominant-negative isoforms of STIM1 and ORAI1, -2 and -3 were provided by L. Birnbaumer and S. Muallem. These cDNAs were driven by CMV promoters in eukaryotic expression plasmids, the coding region fused to eGFP. DN-STIM1 and DN-ORAI1-3 have been shown to specifically interfere with function of their cognate wildtype isoforms and inhibit them. Enzyme-linked immunosorbent assays (ELISAs) for MMP-1, RANTES (regulated on activation, normal T-expressed and secreted), and IP-10 (interferon-γ–induced protein 10 kDa, CXCL10) were conducted using commercially available kits. For MMP-1 secretion, we previously demonstrated its correlation to the specific proteolytic activity of MMP-1 (Li et al. 2009).

Ca^2+^ imaging of BEAS-2B cells was conducted using 2 μM fura-2 acetoxymethyl ester for loading and following a protocol for ratiometric Ca^2+^ imaging using 340/380 nm blue light for dual excitation, recording emissions with specific filter sets. Ratios of the emissions were acquired every 5 sec. ΔR/R_0_ is the fraction of the increase of a given ratio over the baseline ratio, divided by baseline ratio. For stimulation of cells with DEP, we used particles at 100 μg/mL and analyzed only cells with microscopically verified contact with particles. For stimulation with OE, all cells were analyzed. To stimulate TRPV4, hypotonicity was used at 260 mOsmol/L, and 4α-PDD at 10 μM; Ca^2+^ stimulation was accomplished by switching from 0 to 2 mM. Ca^2+^ imaging of primary HBE cells was conducted by excising the air–liquid interface matrix with a scalpel and affixing it to the opening of a glass-bottom dish, with other procedures as for BEAS-2B cells.

### Electrophysiological recordings

Extracellular Ca^2+^ was precipitated by addition of EGTA. We conducted PI3-K Förster resonance energy transfer (FRET) imaging based on FRET of membrane-targeted enhanced green fluorescent protein (eGFP; donor) and the pleckstrin homology domain of Bruton’s tyrosine kinase, fused to mCherry fluorescent protein (acceptor). With low phosphatidylinositol (3,4,5)-trisphosphate (PIP3) levels, the mCherry is cytoplasmic; with increased PIP3 levels, it translocates to the membrane leading to FRET, which we quantified using two-photon fluorescence lifetime imaging. Additional details for electrophysiological recordings are given in Supplemental Material (doi:10.1289/ehp.1002807).

The phosphorylated ERK (phospho-ERK) trafficking assay was performed as described previously (Li et al. 2009). Briefly, BEAS-2B cells were stimulated either with DEP or with OE and fixed (4% paraformaldehyde) at 5-, 10-, 20-, and 30-min time points. Phospho-ERK_1/2_ was verified by fluorescent immunodetection and quantified densitometrically (≥ 75 cells/time point), corrected for background, in the nuclear area using ImageJ software (version 1.42q; Rasband 2009).

Confocal imaging was conducted after immunocytochemical staining for acetylated α-tubulin, PLCβ3, TRPV4, and PAR-2. Fluorescently labeled sections were visualized using a Zeiss LSM710 confocal imaging suite with lasers tuned to the emission spectra of the secondary fluorescent antibodies.

Coimmunoprecipitation studies were conducted using 10^6^ BEAS-2B cells per experiment; cells were harvested in lysis buffer (1% NP40 detergent). Exactly 100 μg protein was incubated with rabbit anti–PLC-β3, rabbit anti-TRPV4, or mouse monoclonal anti–PAR-2 overnight at 4°C, and then solutions were exposed to 15 μL protein-A/G-Sepharose for 4 hr (4°C). After stringency washing, complexes were investigated by Western blotting using antibodies specific for TRPV4, PAR-2, or calmodulin. Normal rabbit or mouse isotype antibodies were used as controls. Western blotting was performed following standard methodology with chemoluminescence detection.

### Statistical analysis

We compared mean and SE of quantified outcome parameters after stimulation with their respective controls. Group comparisons were performed using Student’s *t*-test or analysis of variance with post hoc Scheffe test for multigroup comparison, applying the statistics program StatPlus:mac (AnalystSoft, Vancouver, British Columbia, Canada). Minimum significance was set at *p* < 0.05.

## Results

### The OE of DEP contains the active component to activate *MMP-1*

To understand which component(s) of DEP activate *MMP-1*, we investigated effects of DEP and its OE in human BEAS-2B and primary HBE cells, the latter exposed at air–liquid interface [see Supplemental Material, Figure 1 (doi:10.1289/ehp.1002807)]. Our findings suggest that the carbonaceous core of DEP, by size a carbon nanoparticle, acts as a vehicle carrier for delivery of the highly active, water-insoluble organic fraction to the plasma membrane of human airway epithelia to elicit *MMP-1* activation.

### Extracellular Ca^2+^ influx is necessary for activation of *MMP-1*

Previous studies in lung cells and neurons have shown that particulate matter evokes Ca^2+^ transients (Agopyan et al. 2004); other studies have shown that Ca^2+^ increases activated RAS (Lee and Yasuda 2009) and that DEP activates RAS (Li et al. 2009). Therefore, we examined whether DEP and/or OE causes Ca^2+^ influx and whether this can activate *MMP-1*.

**Figure 1:**
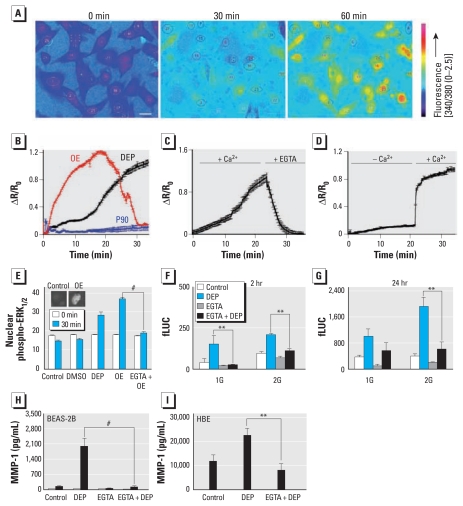
Ca^2+^ influx is required for DEP or OE to evoke MMP-1 secretion. (*A*) Fura-2 Ca^2+^ imaging in BEAS-2B cells exposed to 100 μg/mL DEP at 0, 30, and 60 min (bar = 20 μm). (*B*) Ca^2+^ response (ΔR/R_0_ vs. time) to DEP (*n* = 43), OE (*n* = 62), and P90 control particles (*n* = 48 in BEAS-2B cells). Note the protracted time course of the Ca^2+^ signal, in particular with DEP versus OE. (*C*) Rapid reduction in DEP-induced Ca^2+^ signal in BEAS-2B cells, after the extracellular addition of the Ca^2+^ chelator EGTA (2 mM) (*n* = 32). (*D*) In BEAS-2B cells, response to DEP in the absence or presence of extracellular Ca^2+^; note the rapid increase in fluorescence upon addition of 2 mM CaCl_2_ (*n* = 19). (*E*) Detection of nuclear phospho-ERK_1/2_ in response to DEP and OE depends on presence of extracellular Ca^2+^ (indicated by the lack of response after OE + EGTA treatment in BEAS-2B cells). Note the statistically significant levels of increase for DEP and OE compared with controls (*n* ≥ 75 cells for each condition; 30-min time point); two representative micrographs are depicted as insets. (*F* and *G*) In BEAS-2B cells, DEP-induced transcriptional activation of *MMP-1* depends on external Ca^2+^, as detetermined using 1G and 2G polymorphisms in the firefly luciferase reporter assay (fLUC) at 2-hr (*F*) and 24-hr (*G*) time points; note the statistically significant reduction at the 2-hr time point (*F*) for the –1607GG (2G) polymorphism compared with the –1607G (1G) polymorphism. (*H*) In BEAS-2B cells, DEP-induced secretion of MMP-1 is eliminated in the presence of 2 mM EGTA. (*I*) In HBE cells, DEP-induced secretion of MMP-1 is eliminated in the presence of 2 mM EGTA. Note the different *y*-axis scale in *H* and *I*. **p < 0.01, and ^#^*p* < 0.001.

We found that DEP and OE evoke extracellular Ca^2+^ influx (Figure 1A–D), as indicated by curtailing of the response by addition of EGTA (Figure 1C,D). P90 control carbon nanoparticles had no effect on Ca^2+^ (Figure 1B), whereas DEP activated a uniquely protracted and monotonically increasing response with a peak at approximately 60 min that gradually declined (data not shown). In comparison, the response to OE increased more rapidly, reaching a maximum at approximately 20 min and decreasing to baseline in the next 10 min (Figure 1B), indicating that the particle core retarded Ca^2+^ influx by slowing delivery of the organic fraction to the plasma membrane.

To determine whether DEP-induced Ca^2+^ influx was necessary for transcriptional activation of *MMP-1*, we exposed cells to DEP in the presence and absence of extracellular Ca^2+^ and measured *MMP-1* transcriptional activation at 2 and 24 hr and the appearance of nuclear phospho-ERK at 30 min (Figure 1E–I). These experiments indicated that extracellular Ca^2+^ was necessary for both nuclear translocation of phospho-ERK and *MMP-1* activation in response to DEP or OE. In primary HBE cells, EGTA eliminated MMP-1 secretion in response to DEP or OE (Figure 1H), thus confirming the validity of this mechanism.

We were unable to demonstrate functionality of SOCE in DEP/OE–evoked Ca^2+^ influx [see Supplemental Material, Figure 2A–C (doi:10.1289/ehp.1002807)]. Preincubation with 5 μM thapsigargin or 5 μM GdCl_3_ (Bird et al. 2008) did not markedly change DEP-evoked Ca^2+^ increase. Furthermore, cotransfection of STIM1-DN and ORAI1-3–DN, both known to function in SOCE, did not significantly alter Ca^2+^ responses evoked by DEP or OE.

Finally, we found that DEP and OE also caused the Ca^2+^-dependent secretion of proinflammatory mediators RANTES (Jeffery 2004) and IP-10 (Torvinen et al. 2007) [see Supplemental Material, Figure 2D,E (doi:10.1289/ehp.1002807)]. Thus, in human lung cells, Ca^2+^ influx is necessary for DEP/OE–evoked activation of *MMP-1*, *IP-10*, and *RANTES*.

### PAR-2 is a DEP-sensitive G-protein–coupled receptor (GPCR) that activates G_i/o_, PLCβ3, and PI3-K

**Figure 2:**
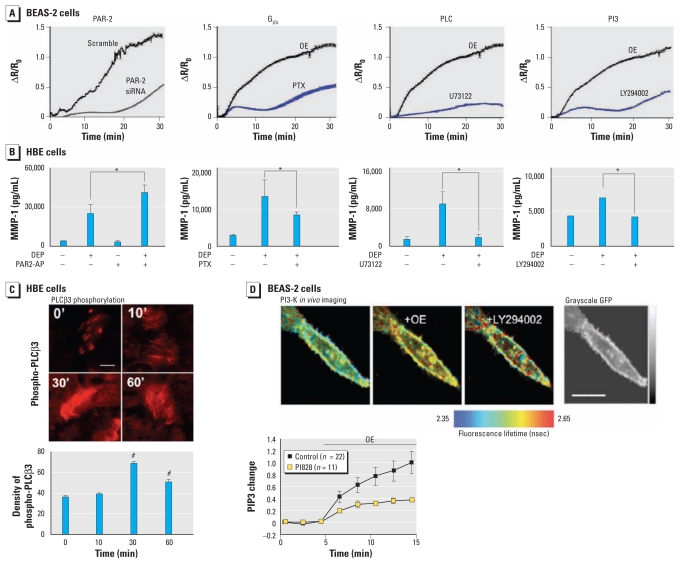
Signal transduction in response to DEP or OE involves PAR-2, G_i/o_, and PLCβ3. (*A*) In BEAS-2B cells, Ca^2+^ response (ΔR/R_0_ vs. time) to OE is significantly attenuated with PAR-2 siRNA [for proof of efficiency, see Supplemental Material, Figure 3A (doi:10.1289/ehp.1002807)], pertussis toxin (PTX; G_i/o_ inhibitor), the PLC inhibitor U73122, and the PI3-K inhibitor LY294002 (*n* ≥ 50 cells per condition). (*B*) In primary HBE cells, modulation of these pathways affects MMP-1 secretion; PAR-2 gain of function potentiates the response, whereas inhibition of G_i/o_, PLC, and PI3-K significantly attenuates/eliminates it. For results in BEAS-2B cells, including PAR-2 gain and loss of function (siRNA), plus all other pathways, see Supplemental Material, Figures 3 and 4 (doi:10.1289/ehp.1002807). (*C*) Increase in phospho-PLCβ3 in response to DEP shown by representative confocal micrographs of immunolabeled primary HBE cells (top; bar = 5 μm) and densitometry (bottom; *n* ≥ 50 cells per time point). Results show the phospho-PLCβ3 increase peaking at 30 min and decreasing at 60 min. (*D*) PI3-K activity in live transfected BEAS-2B cells shown by real-time imaging in response to OE using a novel FRET-based methodology. FRET measurements are shown in micrographs (top; bar = 4 μm), reiterating that PI3-K activity resides in the plasma membrane. The time course of PIP3 generation (bottom) shows that FRET generated by membrane-targeted eGFP and mCherry-tagged pleckstrin homology domain of Bruton’s tyrosine kinase increases robustly after application of OE and became significantly attenuated in response to subsequent application of the specific PI3-K inhibitor LY294002 (10 μM). The graph (bottom) shows the time course of the change in PIP3 evoked by OE (quantified at the membrane) and its attenuation by preincubation with another specific PI3-K inhibitor, PI828 (50 μM). *p < 0.05, and *^#^p* < 0.001 compared with the 0-min time point.

We focused on PAR-2 because an earlier low-throughput proteomics screen revealed that, compared with DEP alone, DEP plus PAR-2–activating peptide (PAR-2-AP) increased MMP-1 secretion (data not shown). We first conducted experiments in BEAS-2B cells by siRNA-mediated knockdown of PAR-2. *PAR-2* mRNA was significantly reduced by PAR-2 siRNA but not by the scrambled control [see Supplemental Material, Figure 3A (doi:10.1289/ehp.1002807)]. Cells treated with PAR-2 siRNA exhibited significantly reduced Ca^2+^ influx, *MMP-1* reporter gene activation, and MMP-1 secretion (Figure 2A; see also Supplemental Material, Figure 3B,C). This demonstrates that PAR-2 functions upstream of Ca^2+^-mediated *MMP-1* activation. In addition to scrambled siRNA controls, PAR-1–specific siRNA had no effect on *MMP-1* activation (data not shown).

Costimulation of BEAS-2B and primary HBE cells with DEP or OE and PAR-2-AP potentiated *MMP-1* activation [Figure 2B; see also Supplemental Material, Figure 3D,E (doi:10.1289/ehp.1002807)]. To boost its moderate expression level in BEAS-2B cells, we overexpressed PAR-2. This led to increased baseline and DEP-evoked *MMP-1* activation, indicating that PAR-2 overexpression is sufficient to increase *MMP-1* expression and to render the cell more responsive to DEP (see Supplemental Material, Figure 3F). Thus, specific activation and inhibition of PAR-2 imply that this receptor is critical in DEP-mediated Ca^2+^ influx that leads to *MMP-1* activation.

We next investigated whether secreted MMP-1 activates PAR-2 proteolytically, as it does for PAR-1 (Boire et al. 2005), which might explain the protracted Ca^2+^ influx in response to DEP. This was not the case, because MMP inhibitors accelerated *MMP-1* reporter gene activity in response to DEP [see Supplemental Material, Figure 3G (doi:10.1289/ehp.1002807)].

We addressed whether β-arrestins are necessary for PAR-2–mediated Ca^2+^ influx in response to DEP or OE (Cottrell et al. 2003). This was not the case in view of Ca^2+^ increase in the absence of β-arrestins 1 and 2 [siRNA-mediated knockdown; see Supplemental Material, Figure 3H (doi:10.1289/ehp.1002807)]. We previously verified elimination of *MMP-1* activation by siRNA-mediated β-arrestin knockdown (Li et al. 2009). Together, these results implicate β-arrestins as MAPK scaffolds necessary for the DEP–MMP-1 response yet dispensable for PAR-2–mediated Ca^2+^ influx in response to DEP or OE.

We examined G_i/o_ signaling because of PAR-2’s known signal transduction mechanisms via this G-protein (Olianas et al. 2007). We found that the DEP–MMP-1 response, namely, Ca^2+^ influx, *MMP-1* transcription, and MMP-1 secretion, depends on G_i/o_, which we targeted specifically with pertussis toxin in both BEAS-2B and primary HBE cells [Figure 2A,B; see also Supplemental Material, Figure 4A (doi:10.1289/ehp.1002807)]. Because G_i/o_ is known to activate PLC (Exton 1996), we next treated cells with PLC-selective inhibitor, U73122, which led to a marked DEP–MMP-1 response (Figure 2A,B; see also Supplemental Material, Figure 4B). PLC has several isoforms; we investigated the β-isoforms because of PLCβ’s link to GPCRs, specifically PLCβ3, in view of its previously established link to G_i/o_ (Senyshyn et al. 1998). When we immunolabeled for PLCβ1–4, we found the most robust expression for PLCβ3 in primary HBE cells (data not shown). Interestingly, using a phospho-specific antibody against PLCβ3, we documented phospho-PLCβ3 up-regulation within 30 min after DEP application (Figure 2C). This finding can help explain the protracted Ca^2+^ influx because PLCβ3, being upstream of extracellular Ca^2+^ influx, was previously demonstrated to be attenuated by phosphorylation (Yue et al. 2000).

Another phospholipid-metabolizing enzyme that signals downstream of G_i/o_ and upstream of TRP channel Ca^2+^ conductances is PI3-K (Zhuang et al. 2004). We identified its critical role in response to DEP or OE using the PI3-K inhibitor LY294002 by documenting significant reduction of Ca^2+^ influx and subsequent *MMP-1* activation in both BEAS-2B and primary HBE cells [Figure 2D; see also Supplemental Material, Figure 4C (doi:10.1289/ehp.1002807)], suggesting the signaling position of PI3-K upstream of Ca^2+^ influx. Moreover, using a novel FRET-based assay, we could visualize the enzymatic activity of PI3-K (change in PIP3) in BEAS-2B cells in response to DEP or OE, which indicated PI3-K activity as an early signaling event (Figure 2D). Furthermore, in addition to time-scale resolution after DEP or OE exposure, this method illustrates the confinement of PI3-K signaling to the plasma membrane.

### TRPV4 forms a DEP-sensitive Ca^2+^ pathway downstream of PI3-K/PLC-β3

**Figure 3:**
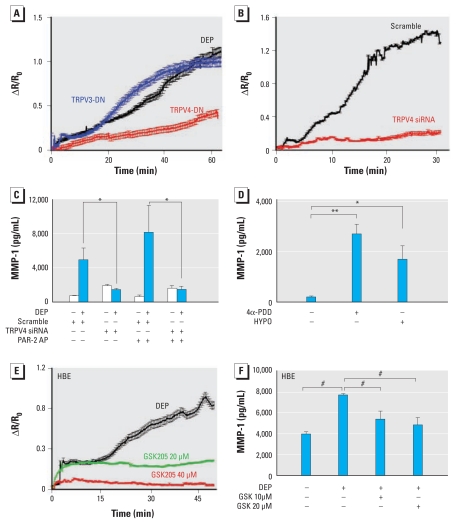
TRPV4 channels are critical for DEP-evoked *MMP-1* activation and function downstream of PAR-2. Abbreviations: +, with; −, without. (*A*) The DEP-evoked Ca^2+^ response in BEAS-2B cells was significantly and specifically attenuated by inhibition of TRPV4 upon transfection of cells with RFP-tagged TRPV4-DN (*n* = 33) and unaffected by transfection with TRPV3-DN [*n* = 44; see also Supplemental Material, Figure 4A (doi:10.1289/ehp.1002807)]. (*B*) TRPV4 loss of function is illustrated by robust inhibition of OE-evoked Ca^2+^ responses in BEAS-2B cells transfected with TRPV4 siRNA compared with transfection with scrambled control siRNA; for proof of efficiency of TRPV4 siRNA, see Supplemental Material, Figure 5B,C. (*C*) siRNA-mediated TRPV4 knockdown in BEAS-2B cells virtually eliminated *MMP-1* activation, thus confirming that TRPV4 functions downstream of PAR-2; note the elimination of MMP-1 secretion in response to DEP or DEP plus PAR-2-AP when TRPV4 is knocked down with TRPV4 siRNA. (*D*) TRPV4 activation is sufficient to activate *MMP-1* hypotonicity (HYPO; 260 mosmol/L) and the specific TRPV4 activator 4α-PDD (10 μM) induced MMP-1 secretion from BEAS-2B cells. (*E*) Specific inhibition of TRPV4 with GSK205 led to dose-dependent reduction of DEP-evoked Ca^2+^ responses in primary HBE cells. (*F*) Specific inhibition of TRPV4 with GSK205 led to a significant reduction of DEP-evoked MMP-1 secretion in primary HBE cells; note the increasing effect with increasing dose. *p < 0.05, **p < 0.01, and *^#^p* < 0.001.

PAR-2 has been shown to sensitize TRP channels, including TRPV1, TRPV4, and TRPA1 (Amadesi et al. 2006; Gatti et al. 2006; Grant et al. 2007). Because TRPV4 is expressed in tracheobronchial epithelia (Lorenzo et al. 2008), we addressed whether it functions downstream of the above signaling cascade, initially by inhibiting its function in BEAS-2B cells expressing TRPV4-DN [see Supplemental Material, Figure 5A–C (doi:10.1289/ehp.1002807)], which produced strong reduction of Ca^2+^ influx in response to DEP yet no reduction for TRPV3-DN (Figure 3A). We also knocked down *TRPV4* using specific siRNA, which effectively down-regulated *TRPV4* mRNA and protein (see Supplemental Material, Figure 5B,C). Compared with the scrambled control, the siRNA-TRPV4 knockdown reduced Ca^2+^ influx in response to DEP or OE (Figure 3B). Thus, TRPV4 is necessary for DEP-evoked Ca^2+^ influx.

Next we addressed whether DEP-evoked, TRPV4-mediated Ca^2+^ influx activates *MMP-1*. Using *MMP-1* reporter assays, we found that TRPV4-specific siRNA decreased *MMP-1* transcriptional activation, thus implying that TRPV4 is critical for DEP/OE–evoked Ca^2+^ influx, which then activates *MMP-1* [see Supplemental Material, Figure 5D (doi:10.1289/ehp.1002807)]. Based on these results, we used the *MMP-1* reporter platform to determine that TRPV4 functions downstream of PAR-2 because siRNA-mediated TRPV4 knockdown virtually eliminated potentiated *MMP-1* activation by DEP or OE plus PAR-2-AP (see Supplemental Material, Figure 5E). This effect of TRPV4-specific siRNA was recapitulated for DEP-evoked MMP-1 secretion in BEAS-2B cells (Figure 3C). Finally, we found that ruthenium red, an unspecific TRP(V) blocker, decreased *MMP-1* activation (see Supplemental Material, Figure 5F).

TRPV4 activation by 4α-PDD or hypotonicity strongly increased MMP-1 secretion, indicating that in airway epithelia, TRPV4 activation is sufficient to up-regulate *MMP-1* (Figure 3D). Furthermore, TRPV4 transfection in BEAS-2B cells increased *MMP-1* reporter activation. Because these findings were obtained in the BEAS-2B cell line, we also tested TRPV4 function in primary HBE cells. First, we were able to significantly attenuate the DEP-evoked Ca^2+^ response by GSK205, a specific small-molecule TRPV4 inhibitor (Phan et al. 2009), in a dose-dependent manner (Figure 3E). In addition, secreted MMP-1 in response to DEP was significantly reduced by two concentrations of GSK205 (Figure 3F). Thus, the cornerstones of TRPV4’s involvement in the DEP–*MMP-1* response, namely, dependence of the Ca^2+^ response and MMP-1 secretion on TRPV4, were recapitulated in primary HBE cells. Whenever possible, we performed the same experiment in human primary HBE cells as in permanent human BEAS-2B cells.

Taken together, these findings point toward critical functioning of TRPV4 in Ca^2+^ influx into human airway epithelia evoked by DEP, a globally relevant air pollutant.

### TRPV4 signaling complex is located on motile cilia of primary human airway epithelia

**Figure 4:**
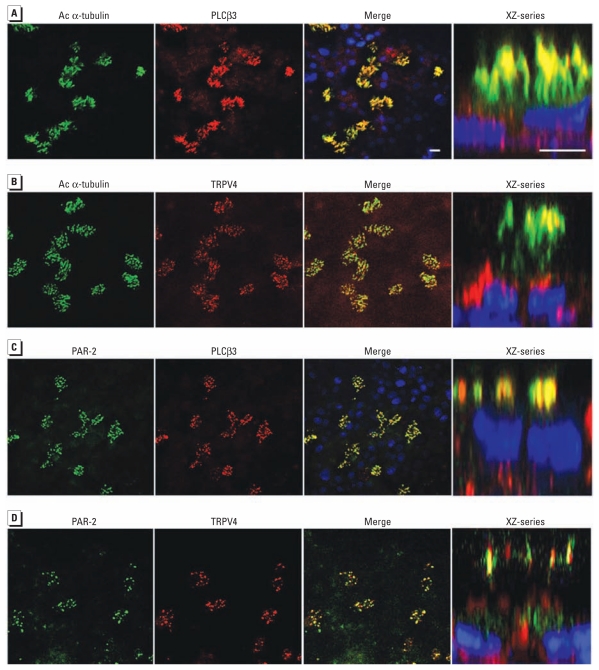
Colocalization of ciliary marker acetylated (Ac) α-tubulin with PLCβ3 (*A*) or TRPV4 (*B*), and colocalization of PAR-2 with PLCβ3 (*C*) or TRPV4 (*D*) in cilia of primary HBE cells. Columns are as follows: green channel, anti-mouse; red channel, anti-rabbit; the merged image; and the the XZ-series reconstruction. Confocal micrographs are top view for the first three columns, and the XZ-series (fourth column) depicts a schematic rendering of an enlarged lateralized section. Bars = 10 μm. Primary HBE cells in *D* were not fully differentiated, showing “budding” cilia at the time of immunolabeling. More elongated cilia were present in *C*, PAR-2 colabeled for PLCβ3, and in *A*, Ac α-tubulin colabeled for PLCβ3. Nevertheless, *A–C* suggest that PAR-2 and TRPV4 colocalize to cilia of primary HBE cells.

Because TRPV4 channels have been found in primary motile cilia of mouse tracheal epithelia (Lorenzo et al. 2008), we determined TRPV4’s subcellular location in human ciliated airway epithelia. Primary HBE cells were differentiated in culture until they became ciliated. They showed ciliary location of TRPV4, PAR-2, and PLCβ3 (Figure 4). Thus, critical DEP-responsive membrane-bound components all localize to motile cilia of primary human HBE cells.

### DEP-facilitated recruitment to a membrane-associated receptor-signaling multicomplex

**Figure 5:**
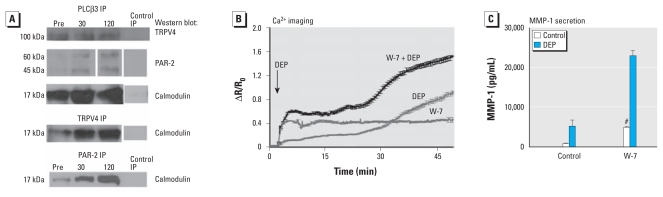
In BEAS-2 cells, protein–protein complex formation after exposure to DEP involves PAR-2, PLCβ3, TRPV4, and calmodulin (which is functional in DEP-evoked Ca^2+^ influx). (*A*) Representative Western blots of immunoprecipitation (IP) experiments performed preexposure (Pre) and 30 and 120 min after DEP exposure; for controls, a control antibody was used for IP. With PLCβ3 IP (top three panels), complexes formed containing TRPV4, PAR-2, and calmodulin; after exposure to DEP, the protein–protein interaction increased for PAR-2 and calmodulin. Under Pre conditions, the PLCβ3–calmodulin and PLCβ3–TRPV4 complexes were present and appreciable. With TRPV4 IP, complexes formed containing calmodulin; after DEP exposure both interactions clearly increase. PAR-2 IP shows that PAR-2 forms a protein–protein complex with calmodulin and that this interaction increased after DEP exposure. Potentiating effect of the specific calmodulin inhibitor W-7 on DEP-evoked Ca^2+^ influx (*B*) and MMP-1 secretion (*C*). The arrow in *B* indicates the time of DEP exposure. *^#^p* < 0.001 for W-7 DEP compared with control DEP and for W-7 control compared with control control.

Given the ciliary localization of the DEP-evoked transduction cascade, we asked whether these membrane-associated signaling molecules coaggregate in response to DEP or OE (Figure 5). In aggregate, DEP-responsive membrane-bound signaling is characterized by a nonincremental interaction between PLCβ3 and TRPV4, with subsequent recruitment of PAR-2 and calmodulin caused by DEP exposure. Calmodulin’s response to DEP is inhibitory, because specific inhibition of calmodulin with W-7 increased Ca^2+^ signaling and *MMP-1* activation (Figure 5B,C). This suggests as explanatory mechanism(s) an increase in intracellular Ca^2+^ concentration ([Ca^2+^]*_i_*) via disinhibited TRPV4 and/or activation of PLCβ3, both of which have previously been shown to bind calmodulin.

### COPD-associated TRPV4_P19S_ increases *MMP-1* activation in response to DEP or OE

For human *TRPV4*, a number of genetic polymorphisms enhance susceptibility for COPD; one of them, P19S, is located in the coding region (Zhu et al. 2009). In another study, TRPV4_P19S_ was reported as a DN channel in transfected HEK cells in response to weak but not strong hypotonicity (Tian et al. 2009). Because our finding that DEP-evoked Ca^2+^ influx via TRPV4 causes *MMP-1* activation rationalizes airway injury by MMP-1 as caused by TRPV4 channel activity, not by DN channels, we attempted to resolve these seemingly contradictory concepts.

**Figure 6:**
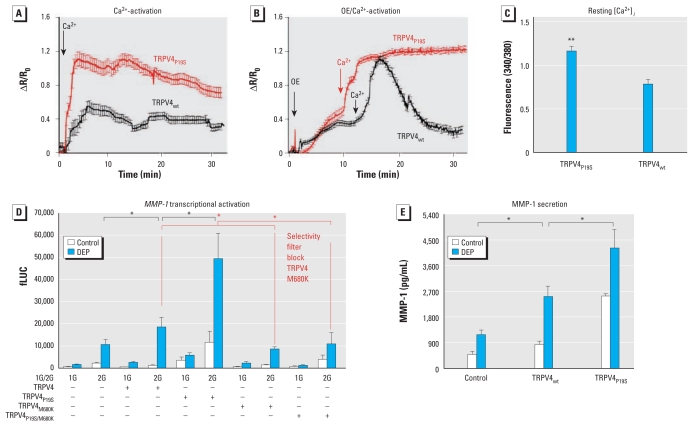
In BEAS-2B cells, TRPV4_P19S_ functions as a gain-of-function channel in response to DEP, causing increased *MMP-1* activation via influx of Ca^2+^. (*A*) Time course showing increased Ca^2+^-facilitated activation of TRPV4_P19S_ compared with TRPV4_wt_ (*n* ≥ 24 cells per group). (*B*) Time course showing OE-evoked Ca^2+^ influx in Ca^2+^-free buffer (note the presence of Ca^2+^ in OE) followed by the addition of external Ca^2+^ (2 mM), which leads to accelerated Ca^2+^ influx for TRPV4_P19S_ versus TRPV4_wt_. The signal of TRPV4_wt_ declined after the peak, whereas TRPV4_P19S_-transfected cells did not desensitize; the signal remained high at least during this observation period. Arrows in *A* and *B* indicate the time of Ca^2+^ or OE exposure. (*C*) Internal [Ca^2+^]*_i_* was significantly elevated in TRPV4_P19S_-expressing BEAS-2B cells cultured in external media containing Ca^2+^. (*D*) In keeping with Ca^2+^ responses shown in *B*, TRPV4_P19S_ increased *MMP-1* transcriptional activation. First, transfection of TRPV4_wt_ increased *MMP-1* reporter gene activation in response to OE by approximately 2-fold. Second, transfection of TRPV4_P19S_ strikingly increased baseline *MMP-1* reporter gene activation in response to OE by a factor of approximately 4–5 versus control-transfected cells and by a factor of > 2 versus TRPV4_wt_. TRPV4_wt_- and TRPV4_P19S_-mediated increases were virtually eliminated with a second point mutation, M680K (selectivity-filter block). This finding indicates that the effects of TRPV4_wt_ and TRPV4_P19S_ are mediated by an influx of external Ca^2+^ through the channel’s pore. (*E*) Validity of results and conclusions for MMP-1 secretion by transfected BEAS-2B cells. *p < 0.05, and **p < 0.01.

Compared with wild-type TRPV4 (TRPV4_wt_), TRPV4_P19S_ exhibited gain-of-function effects in Ca^2+^ influx, patch clamp, *MMP-1* reporter gene activation, and MMP-1 secretion [Figure 6; see also Supplemental Material, Figure 6 (doi:10.1289/ehp.1002807)]. For *MMP-1* transcriptional activation, TRPV4_P19S_ gain-of-function effects were strictly dependent on Ca^2+^ influx. This was evidenced by inhibitory effects of TRPV4_P19S/M680K_, where the selectivity-filter– blocking M680K mutation causes Ca^2+^ impermeability, leading to elimination of gain of function (Figure 6D). Furthermore, Ca^2+^ influx in response to changes in Ca^2+^ concentration and to DEP or OE were significantly increased in TRPV4_P19S_ versus TRPV4_wt_, as was nonstimulated [Ca^2+^]*_i_* [extracellular Ca^2+^ concentration, 2 mM) (Figure 6A–C; see also Supplemental Material, Figure 6). Thus, in a human airway epithelium–derived cell line with robust similarity to primary HBE cells, TRPV4_P19S_ functions as Ca^2+^-permeable gain-of-function channel to hyperactivate the pathogenic mediator gene *MMP-1* in response to the common air pollutant DEP.

## Discussion

**Figure 7:**
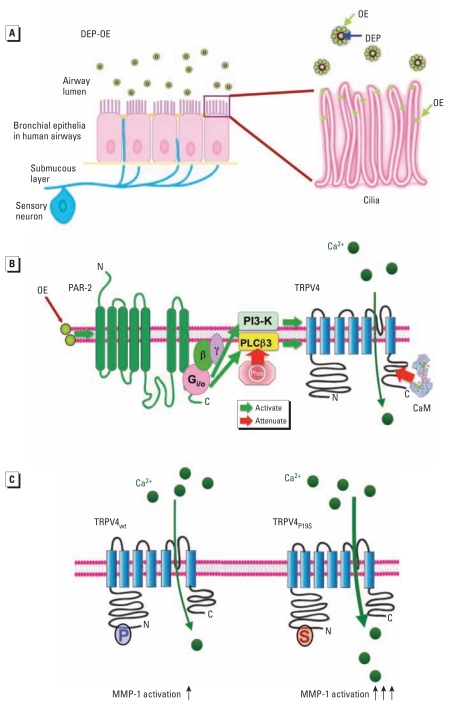
Schematic overview showing effects of DEP on signaling in human ciliated airway epithelia leading to TRPV4-mediated Ca^2+^ influx. Abbreviations: β, G-protein β; γ, G-protein γ; C, C-terminus of TRPV4; N, N-terminus of PAR-2; P, position 19 of TRPV4 ion channel protein proline (wild-type); S, position 19 of TRPV4 ion channel protein proline serine (P19S polymorphism). (*A*) Overview of respiratory epithelia exposed to airborne DEP. (Left) Apical DEP with attached OE approaching the ciliary brush, basement membrane, and innervating nerve endings (blue). (Right) Detailed view of cilia showing DEP core particles contacting cilia and delivering organic chemicals (OE; light green circles) to the plasma membrane (ciliary colocalization is shown in Figure 4). (*B*) The signaling cascade begins with activation of PAR-2 (green); this ultimately leads to the influx of Ca^2+^ (dark green circles) via TRPV4 (blue) by GPCR signaling encompassing G_i/o_, which in turn leads to activation of PLCβ3 and PI3-K. PLCβ3 is phosphorylated (Phos) in response to DEP, partially accounting for the protracted Ca^2+^ response. PLCβ3 and PI3-K then regulate Ca^2+^ influx through TRPV4, which binds calmodulin (CaM), which is enhanced by DEP exposure; the increased CaM also protracts Ca^2+^ influx. (*C*) TRPV4-mediated Ca^2+^ entry activates RAS-RAF-MEK MAPK signaling (Li et al. 2009), resulting in reprogramming of transcriptional mechanisms that orchestrate remodeling of the extracellular matrix via activation of *MMP-1*. The COPD-susceptibility polymorphism TRPV4_P19S_ functions as a gain-of-function channel for additional Ca^2+^ influx and *MMP-1* activation, thus being relevant to human health.

We identified a novel DEP-activated signaling pathway in human airway epithelia that consists of a GPCR (PAR-2) signaling to a TRP channel (TRPV4). Specific activation of PAR-2 by OE leads to Ca^2+^ influx mediated by TRPV4 channels, via membrane phospholipid signaling involving PLCβ3 and PI3-K. This pathway can be used to develop effective medical therapy for human airway injury caused by airborne particulate pollution, a well-recognized global health problem that contributes to development of COPD and other respiratory illnesses. Regarding COPD, we identified a molecular, cellular, and subcellular mechanism for how the COPD-predisposing nonsynonymous genetic polymorphism TRPV4_P19S_ may potentiate DEP-evoked Ca^2+^ influx and activation of airway-pathogenic *MMP-1.* A schematic of our findings is presented Figure 7.

In the context of our results, three qualifiers should be mentioned. First, TRPV4 may not be the only critical Ca^2+^ conductance that is activated. Second, regarding generation of phospholipid molecules with modulatory activity on TRPV4 (e.g., by PI3-K and PLCβ3), we speculate that ratios of active small molecules in the immediate vicinity of the channel are critical for channel function. These two aspects deserve further study. Third, mice do not have an *MMP-1*–orthologous gene, thus restricting the direct translatability of our findings into a rodent model that is amenable to genetic engineering.

The critical participants of the DEP response, PAR-2, PLCβ3, and TRPV4, were localized to motile cilia of differentiated primary human respiratory epithelia. Motile cilia represent a cellular extension that enhances transfer of DEP organics to ciliary membranes via aerogenic exposure, given the enormous increase in cellular surface (Figure 7A). This delivery mechanism can be viewed as “slow-release” delivery of organic chemicals from coated particles to lipid membranes.

We also intend to address the question of how this novel signaling pathway has been shaped by evolution. PAR-2 signaling, as yet another variant of sensory signaling (Schmidlin and Bunnett 2001), has evolved in respiratory cilia likely as a sentinel for activation by microbial proteases, such as granzyme and chitinase. PAR-2 signaling thus would have conferred survival benefits in host defenses of airway integrity yet has subsequently been hijacked by man-made air pollution, a detrimental turn that occurred very recently, during the last milliseconds of the evolutionary clock.

A recently published landmark report showed that bitter taste receptors and PLCβ2 localized to motile cilia of human primary airway epithelia (Shah et al. 2009); however, we found a different isoform, PLCβ3, that localized to respiratory cilia. Whether bitter taste receptor–mediated Ca^2+^ increase will trigger a different set of responses compared with PAR-2/TRPV4–mediated Ca^2+^ influx remains to be established. Our results in the present study suggest that TRPV4-mediated Ca^2+^ influx in response to DEP or OE leads to maladaptive, propathogenic reprogramming of gene-regulatory mechanisms in human airway epithelia.

In the novel signaling mechanism in human airway epithelia described here, Ca^2+^ influx is characterized by uniquely slow kinetics. Two possible causes for the slow kinetics are *a*) DEP-dependent PLCβ3 phosphorylation, and *b*) DEP-enhanced calmodulin binding to a receptor-signaling multiplex containing TRPV4 and PLCβ3 (Figures 5, 7), both of which attenuate signaling via known properties of the modified phospholipase or channel. The attractive hypothesis of proteolytic activation of PAR-2 by MMP-1 has not been not corroborated.

This study is relevant for global human health because of the global presence of DEP. However, we also discovered a possibly novel mechanism of airway injury that is caused by DEP yet enhanced by the human COPD-susceptibility polymorphism TRPV4_P19S_. Our identification of TRPV4_P19S_ as a gain-of-function Ca^2+^-permeable channel in a human respiratory epithelial cell line, in response to DEP, links COPD pathogenesis to pathologically increased Ca^2+^ influx into human airway epithelia elicited by a globally relevant air pollutant. Furthermore, our results imply that two human genetic polymorphisms are linked to respiratory health, TRPV4_P19S_ and *MMP-1* (−1607G/GG), thus highlighting the concept of disease susceptibility as a function of genetic “makeup” combined with environmental insults. Finally, we note yet another translational medical implication: The novel pathway described here can be targeted by inhalation of compounds that can specifically inhibit critical signaling molecules. In other words, although DEP injures respiratory epithelia via a luminal–apical unloading mechanism of DEP organics delivered by carbonaceous nanoparticles, this very same route could become the avenue for safe and effective therapy now that key participants are known.
